# Retraction Note: The effects of intermittent bolus paravertebral block on analgesia and recovery in open hepatectomy: a randomized, double-blinded, controlled study

**DOI:** 10.1186/s12893-024-02480-6

**Published:** 2024-06-17

**Authors:** Jin Wang, Xulei Cui, Yuelun Zhang, Xinting Sang, Le Shen

**Affiliations:** 1 Department of Anesthesiology, Peking Union Medical College Hospital, Chinese Academy of Medical Sciences, Beijing, China; 2 Center Research Lab, Peking Union Medical College Hospital, Chinese Academy of Medical Sciences, Beijing, China; 3 Department of Hepatology, Peking Union Medical College Hospital, Chinese Academy of Medical Sciences, Beijing, China; 4 State Key Laboratory of Complex Severe and Rare Disease, Beijing, China

**Retraction Note: BMC Surgery (2023) 23:218**

10.1186/s12893-023-02125-0

The authors have retracted this article. After publication, concerns were raised that there was overlap between this randomized controlled trial and a retrospective cohort study reported by the authors previously [1]. Investigation by the authors found that there were inaccuracies in the reporting of the time frame of the retrospective cohort study [1] and that some patient data from [1] were also included in the reporting of the outcome of this randomized controlled trial in error. The authors apologise for the inconvenience. All authors agree to this retraction.
